# Weapon Violence Dataset 2.0: A synthetic dataset for violence detection

**DOI:** 10.1016/j.dib.2024.110448

**Published:** 2024-04-20

**Authors:** Muhammad Shahroz Nadeem, Fatih Kurugollu, Hany F. Atlam, Virginia N.L. Franqueira

**Affiliations:** a School of Technology, Business and Arts, University of Suffolk, Ipswich IP4 1QJ, United Kingdom; b College of Computing and Informatics, Department of Computer Science, University of Sharjah, Sharjah, 27272, United Arab Emirates; c Cyber security Centre, Warwick Manufacturing Group(WMG), University of Warwick, Coventry CV4 7AL, United Kingdom; d School of Computing, University of Kent, Canterbury CT2 7NZ, United Kingdom

**Keywords:** Synthetic virtual violence, WVD, Violence detection, GTA-V, Hot and Cold weapons

## Abstract

In the current era, satisfying the appetite of data hungry models is becoming an increasingly challenging task. This challenge is particularly magnified in research areas characterised by sensitivity, where the quest for genuine data proves to be elusive. The study of violence serves as a poignant example, entailing ethical considerations and compounded by the scarcity of authentic, real-world data that is predominantly accessible only to law enforcement agencies. Existing datasets in this field often resort to using content from movies or open-source video platforms like YouTube, further emphasising the scarcity of authentic data. To address this, our dataset aims to pioneer a new approach by creating the first synthetic virtual dataset for violence detection, named the Weapon Violence Dataset (WVD). The dataset is generated by creating virtual violence scenarios inside the photo-realistic video game namely: Grand Theft Auto-V (GTA-V). This dataset includes carefully selected video clips of person-to-person fights captured from a frontal view, featuring various weapons—both hot and cold across different times of the day. Specifically, WVD contains three categories: Hot violence and Cold violence (representing the violence category) as well as No violence (constituting the control class). The dataset is designed and created in a way that will enable the research community to train deep models on such synthetic data with the ability to increase the data corpus if the needs arise. The dataset is publicly available on Kaggle and comprises normal RGB and optic flow videos.

Specifications Table

**Table:** 

Subject	Computer Vision and Pattern Recognition, Multimedia, Artificial Intelligence
Specific subject area	Violence classification and detection
Data format	Synthetic Raw
Type of data	RGB and Optic Flow videos(.avi)
Data collection	The process of data generation involves the recording of frames within a simulated setting. The chosen virtual environment for this endeavour is Grand Theft Auto V (GTA-V). Within this platform, scenarios depicting instances of violence were meticulously crafted through the modification (“modding”) of the game. Subsequently, images capturing these scenarios were procured using specialized software and subsequently assembled into a cohesive dataset. Prior to the conversion of images into video format, a thorough manual human visual inspection was conducted, accompanied by preprocessing procedures. Furthermore, in conjunction with the dataset, the Dense Optic Flow was computed from the acquired frames. This additional optical flow information, calculated through the Dense Gunnar-Farneback method, is an integral component of the dataset and is made publicly accessible.
Data source location	Institute: University of Derby, College of Engineering and Technology, University of Derby, Markeaton Street, Derby DE1 1DW, United Kingdom
Data accessibility	Repository name: Kaggle Data identification number: 10.34740/kaggle/dsv/2535579 Direct URL to data: https://www.kaggle.com/datasets/thelarka/weapon-violence-dataset-wvd Instructions for accessing these data: Go to the provided link and simply click the download button at the top right corner.
Related research article	Nadeem, M.S., Franqueira, V.N.L., Kurugollu, F., Zhai, X. (2019). WVD: A New Synthetic Dataset for Video-Based Violence Detection. In: Proceedings of the 39th International Conference on Innovative Techniques and Applications of Artificial Intelligence (SGAI 2019), pp. 158–164. https://doi.org/10.1007/978-3-030-34885-4_13

## 1 Value of the Data


 

1. Ethical and Moral Neutrality: The dataset avoids ethical and moral implications, eliminating concerns related to the preservation of privacy for individuals engaged in simulated fighting scenarios.
2. Psychological Impact Mitigation: Synthetic data usage shields individuals from unnecessary exposure to distressing content, alleviating concerns about potential psychological impacts on research participants.
3. Open Accessibility: The dataset can be publicly shared and disseminated to third parties without necessitating consent from governmental entities, promoting transparency and collaborative research efforts.
4. Enhanced Visual Realism: The synthetic nature of the data allows for the inclusion of extensive depictions of weapons, blood, and gory scenes, surpassing limitations associated with real-world data collection.

## 2 Background

Violence detection, a computer vision task, is fraught with ethical and moral implications. The majority of open-source datasets for violence comprise data sourced from Hollywood movies or video repositories like YouTube. Accessing real datasets from law enforcement agencies is virtually impossible, thus severely limiting the existing datasets in terms of the extent of violence that can be depicted and shared with the research community. This challenge is compounded by the inherent inability to share such data with third parties, primarily due to the presence of graphic content such as gore, blood, and weapons. In many cases, maintaining the privacy of individuals involved in these violent acts is imperative. As a result, generating large-scale annotations, which is already an expensive and labor-intensive task, becomes problematic [1]. These annotated datasets are crucial for supervised learning algorithms as deep learning algorithms require substantial amounts of data. The acquisition of a large number of violence samples, which are rare events, further complicates this landscape [2]. The literature suggests that various synthetic datasets have been generated for diverse research problems using simulated environments [3]. To tackle challenges in computer vision for violence analysis, a synthetic dataset named the Weapon Violence Dataset (WVD) is proposed.

## 3 Data Description

**Fig. 1:**
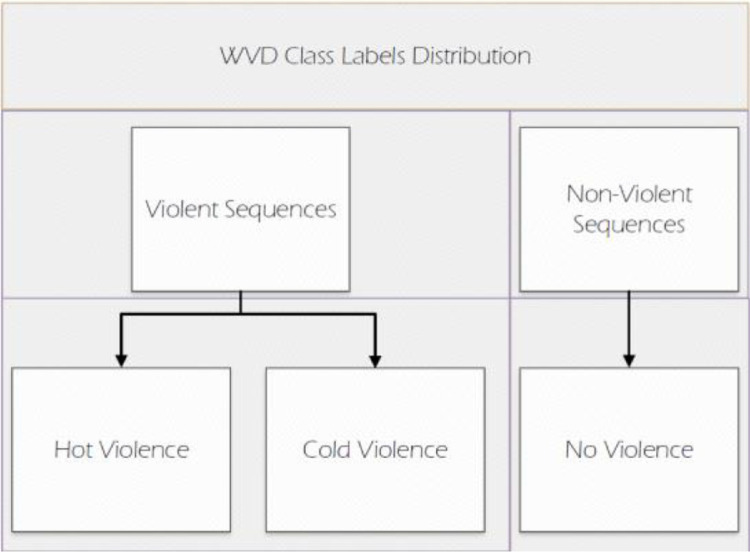
WVD with three classes: Hot, Cold, and No Violence, allowing two of these to form the conventional two-label system for violence classification: Violence vs. No Violence.

The WVD stands as a pioneering synthetic dataset for violence. It is crafted by harnessing the immersive photorealistic environment of the open-world game, Grand Theft Auto V (GTA-V) [4]. It must be noted that GTA-V has been utilized to generate synthetic datasets for other computer vision problems. This platform is well established in synthetic data generation, however, to the best of our WVD is the only synthetic violence dataset specifically created using GTA-V. Rockstar Games allows public modders to modify the game's platform making it a virtual sandbox. Through writing custom codes based on the games API the developers can modify all the programming aspects of the game. This functionality has enabled researchers to utilize this game for research purposes. This aspect of the game is harnessed to generate scenes, assign weapons, and define combat styles for Non-Player Characters (NPCs), thereby creating all the data samples in the WVD for both violent and non-violent scenes. The class distribution of WVD is shown in Fig. 1. The dataset has three classes in total, Hot Violence, Cold Violence, and No Violence. The Hot and Cold are both violent classes, except for different weapons being utilised in the fights. No violence class consists of normal action performed by NPCs.

**Hot weapons are defined as**: ***“Firearms that use gunpowder”*.**

**Cold weapons are defined as**: ***“Traditional weapons with no gunpowder usage”.***

In each scene, NPCs are present, with weaponry exclusively assigned to one NPC. This allocation designates the role of the aggressor, while the other NPC assumes the role of the victim and is consequently devoid of any weaponry. However, it must be emphasised, that even with no weapon, the victim NPC can and, in some samples, does successfully fight back when attacked. Table 1, Table 2 list the in-game “Hot” and “Cold” weapons respectively. The “No Violence” class acts as a control class, both the NPCs are in a non-aggressive state and thus performing their normal assigned actions. There are numerous more non-violent actions that can be performed in the game. Table 3 shows all the in-game non-violent actions that the NPCs perform in the WVD.

**Table 1:** The list of hot weapons used in the dataset (Game name followed by real name).

Hot Weapons		
AP Pistol (OTs-33 Pernach or HK Mark 23)	Combat Pistol (HK P2000)	Pump Shotgun (M590A1)
Bullpup Rifle (QBZ-95)	Carbine Rifle (HK416, LR-300)	Assault Shotgun (UTAS UTS-15)
Micro SMG (IMI Uzi)	SMG (HK MP5)	MG (PK)
Combat MG (M249)

**Table 2:** The list of cold weapons used in the game.

Cold Weapons		
Baseball Bat	Broken Bottle	Crowbar
Hammer	Hatchet	Knife
Pipe Wrench	Machete	Golf Club

**Table 3:** The list of no violence actions.

No Violence Activities		
Argument	Car Repair	Construction
Conversation	Exercise	Gardening
Getting Arrested	Dancing	Yoga

Each of these video sequences once designed and fixed can be run multiple times. Specifically for violent scenes, each time the fight begins, the game's AI takes control of the NPCs. Consequently, each fight sequence is distinct and unique, featuring completely different fight choreography. This suggests that even within the same scene (featuring identical NPCs), the nature of the fight will vary. This observation is evident in each sample of the WVD, where the fights occur under diverse lighting conditions. Table 4 shows the times of the day in which each violent and non-violent sequence is captured.

**Table 4:** Time settings for which video sequences are recorded.

Times of the Day		
Morning	Dusk	Midday
Sunset	Midnight	Afternoon

GTA-V offers the distinct advantage of a robust real-world combat engine, enabling NPCs to autonomously engage in combat without human oversight. The game's default engine encompasses diverse fighting styles, authentic weapon gestures for loading and reloading, defensive maneuvers, and the capability to take cover when under attack. These features are observable in videos of the WVD. While the game allows for the addition and control of multiple other parameters, this version of the dataset exclusively focuses on person-to-person violence scenes.

**Fig. 2:**
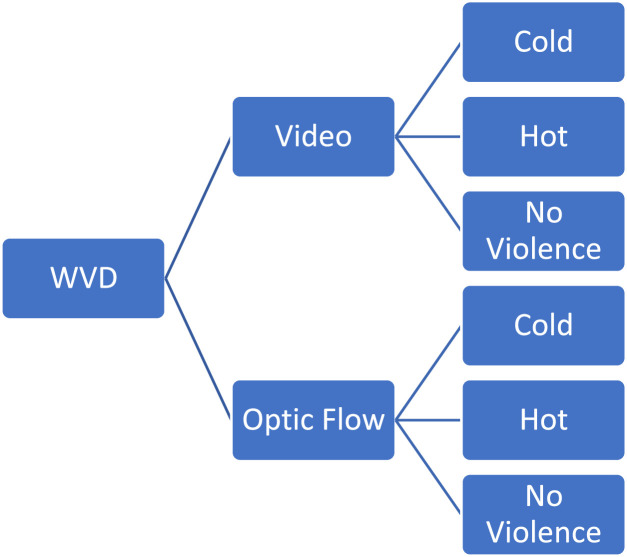
Folder structure of WVD on Kaggle.

The dataset is openly accessible on Kaggle, complete with the mod and installation files. Fig. 2 presents a detailed breakdown of the dataset's folder structure, organized by classification. Within the WVD, a primary video folder contains RGB videos depicting various fight sequences. Furthermore, the WVD includes a folder specifically dedicated to motion imagery derived from the fights, located within the Optic Flow directory.

The calculation of optic flow is performed using the Dense Gunnar-Farneback method [5], a widely utilized technique in computer vision for motion estimation. Additionally, a small video is provided to demonstrate how the scenarios are set up and executed within the game.

**Fig. 3:**
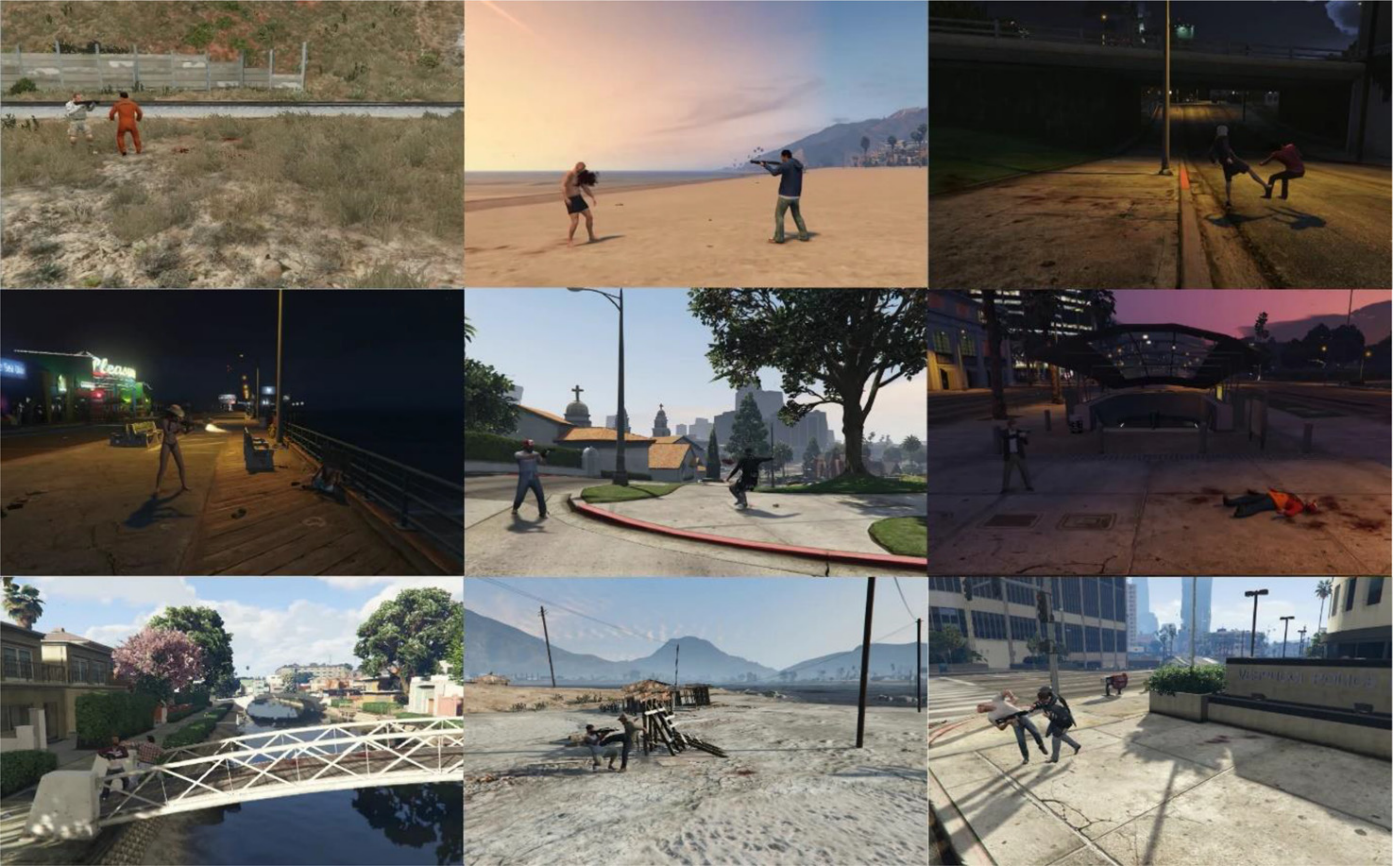
Frame sequences capturing fights with hot weapons.

**Fig. 4:**
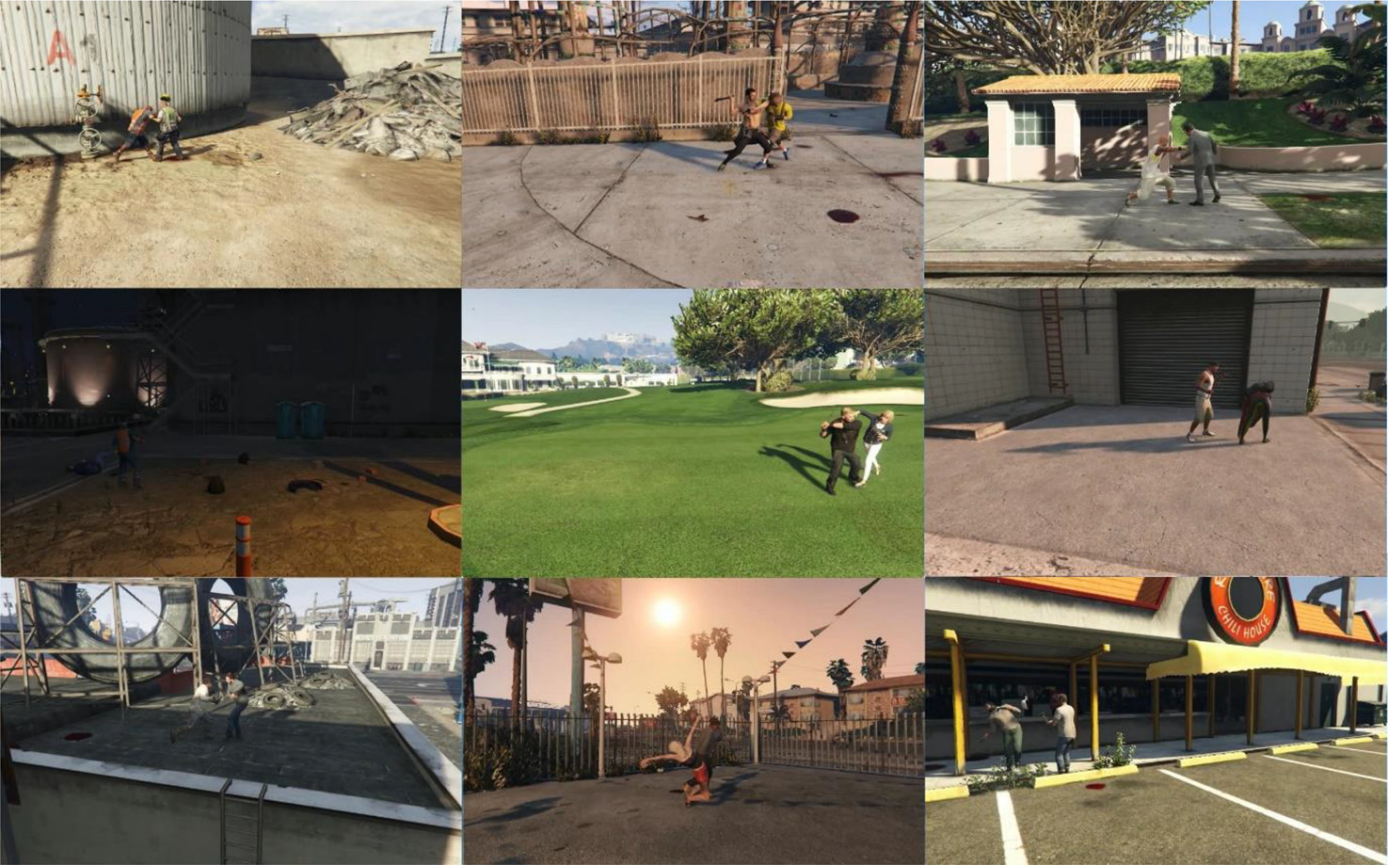
Frame sequences capturing fights with cold weapons.

**Fig. 5:**
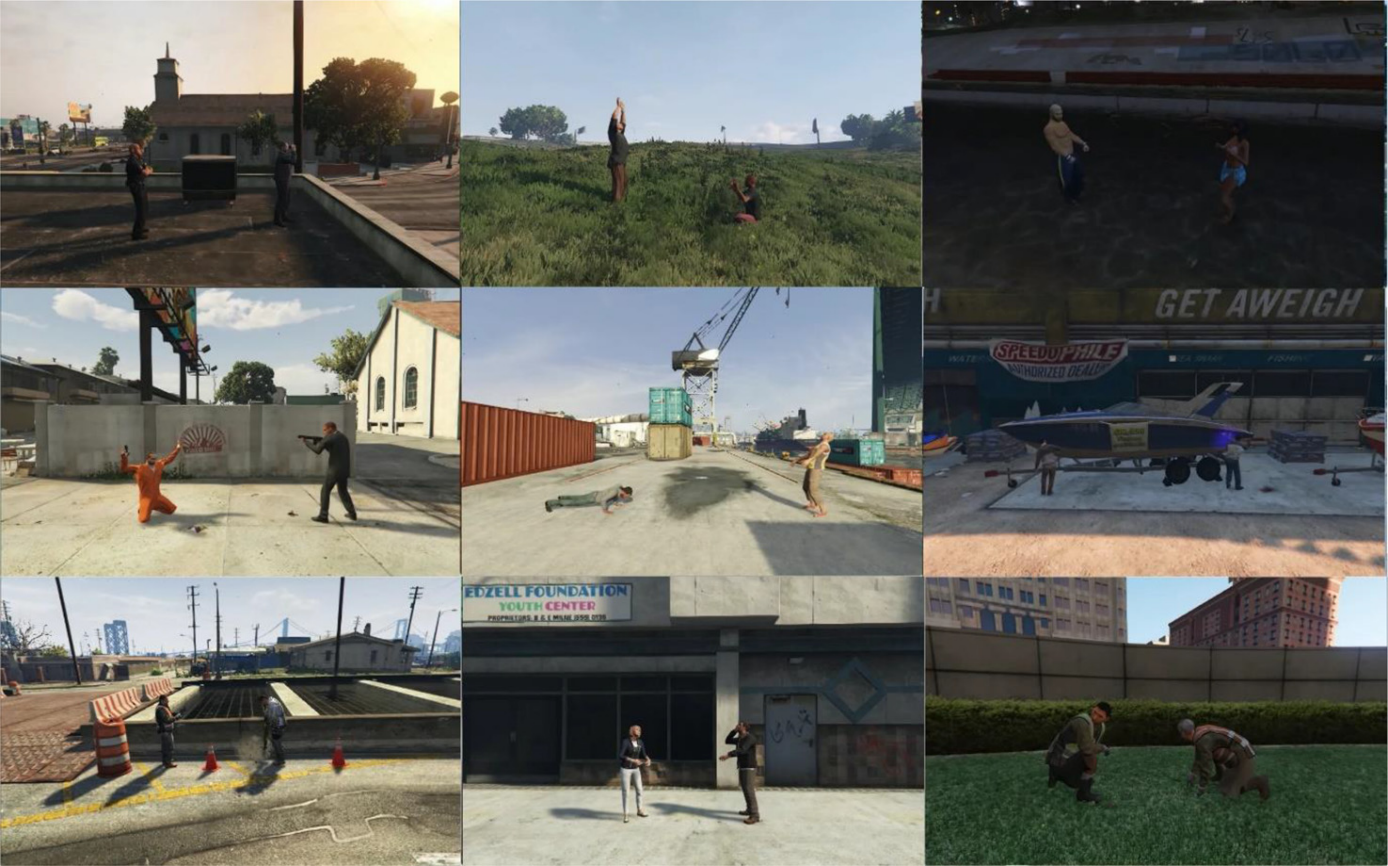
Frame sequences capturing No Violence scenes.

The dataset consists of a total of 334 videos, divided into 60 instances of hot violence, 54 instances of cold violence, and 54 instances categorized as no violence for both RGB and optic flow data. Each video maintains a resolution of (800 × 500) pixels and adheres to a frame rate of 15 frames per second. The duration of these fight scenes ranges from 10 to 72 s. It is noticeable that fights involving hot weapons have, on average, shorter durations due to the involvement of lethal weapons, averaging approximately 15 s. Conversely, cold fights tend to be longer, with an average duration of 45 s. Fig. 3 illustrates instances of violence captured at different times of the day, prominently featuring instances of firearm discharges. Similarly, Fig. 4 depicts distinct action scenes involving cold weapons. In Fig. 5, all nonviolent actions, including certain sequences within the grey area such as the arrest scene featuring instances of weapon use, are observable.

Keeping in mind the advantages of synthetic virtual datasets, WVD enjoys the following advantages over traditional violence detection datasets.

1. No ethical implications or moral objections to violence in a virtual world.
2. Complete control over the environment settings.
  a. Multiple camera angles (WVD has frontal view)
  b. Different combinations of daytime, weather, and climate settings.
  c. Huge arsenal of weapons.
  d. Customisable NPCs: in terms of appearance, gender, and behavioural aggression.
3. Ability to control a huge number of parameters for the virtual setup.
4. Ability to incorporate people-object interactions.
5. Ability to overall generate huge amounts of data including the rarely occurring scenarios.
6. Ability to programmatically generate class labels, reducing human labour (automatic annotation) [6].
7. No manual human effort is required in the execution of the violent scenario.
8. Increased flexibility to shift from person-to-person fights to crowd based violence.
9. Ability to generate high/low resolution imagery of violent sequences based on requirements.
10. Ability to regenerate, reuse and save virtual violence setup.
11. Low-cost solution to train deep networks.
12. Ability to maintain anonymity, while recreating real world violence scenes, without compromising the identities of real people.

## 4 Experimental Design, Materials and Methods

**Fig. 6:**
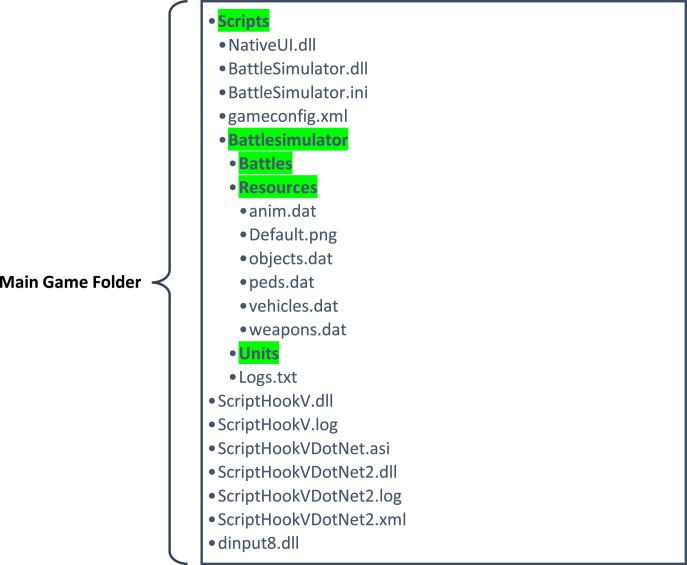
Folder structure of GTA-V, highlighting folders in green. The remaining files are necessary for successful mod installation.

The synthetic video data of the Weapon Violence Dataset (WVD) is generated using the GTA-V game. The initial step involves obtaining a licensed copy of the game through the Steam gaming platform. Steam installs games in the Windows directory, facilitating users to add mods (code files) in specific folders to modify and manipulate the gameʼs virtual environment according to their preferences. This capability has fostered a thriving modification community, making GTA-5 a valuable resource for computer vision research. For the WVD, an open-source mod known as the “Battle Simulator” [7] is utilized. Installation of this mod involves copying the files as depicted in Fig. 6. The mod files are placed within the scripts folder, while the Script Hook files are placed in the main game folder.

**Fig. 7:**
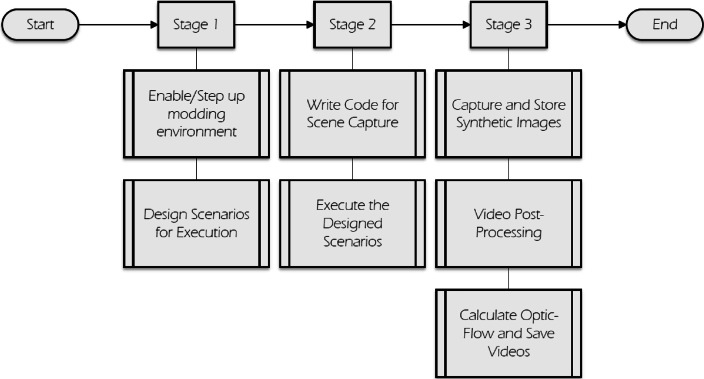
The three-stage process to develop the synthetic images for the proposed WVD dataset.

The overall process of designing, generating, and collecting data for the WVD can be segmented into three main stages. Fig. 7 illustrates these stages along with their respective sub-stages.

The experimental setup utilizing GTA-V allows for the design and capture of violence scenarios, followed by preprocessing, consolidation, and combination of these scenarios to create a comprehensive dataset.

**Stage 1:** The initial step involves installing the mod in the game and ensuring that the folder structure aligns with the configuration depicted in Fig. 6. Once this setup is complete, the subsequent task in stage 1 is the designing of scenarios. Given that there are three classes in the WVD—namely, “Hot Violence,” “Cold Violence,” and “No Violence”—scenarios need to be configured for each of these classes. This task necessitates a one-time human effort, involving the meticulous design of the fight segments. This includes selecting the two NPCs, determining their appearance, assigning weapons or tasks, setting up the background environment, and configuring daytime settings, among other considerations.

**Stage 2:** The designed scenarios from Stage 1 are executed and captured in Stage 2. Since the experimental method leverages game AI, no human intervention is necessary during the duration of the fights. The game engine determines the duration, outcome, and overall manner in which the NPCs engage in combat. This approach also yields unique positions and depths for the NPCs. Executing each scenario multiple times results in distinct fights, with potential variations in outcomes. However, the game's AI tends to Favor the NPC wielding a weapon, increasing the likelihood of incapacitating the opposing party. The fights are autonomous, making each fight scene unique. However, for the nonviolent class, actions are repetitive and monotonic in nature, adhering to a predetermined set of motions defined by the game engine. This characteristic establishes a control class. The maximum utilization of GTA-V's capabilities in the experimental method yields raw footage depicting both violence and nonviolence.

**Stage 3:** The captured images undergo post-processing, beginning with a manual inspection by human observers to detect any potential faults in the capture process. This may involve identifying issues such as code malfunction, blank images, or scenarios disrupted by game crashes. Once these visual anomalies are swiftly addressed, unwanted frames are removed from each captured instance of data, particularly those occurring after the conclusion of fights. Frames depicting the victorious NPC celebrating or taunting are eliminated, as such behaviour is not characteristic of real-world scenarios involving public weapon-based violence. Subsequently, the captured data is aggregated and converted into Audio Video Interleave (.AVI) format to conserve storage space. Additionally, motion information is calculated using the Gunnar-Farneback algorithm [5], with the resulting Dense Optic-Flow motion integrated into the WVD. This step is particularly crucial as many multi-modal deep learning methods combine motion information to enhance the performance and confidence of their models. Therefore, this enhancement was included in the methodology**.**

## Limitations

The limitations of WVD are mostly associated with the shortcomings that come from synthetic data usage. Even though with numerous advantages of synthetic data, it does come with certain limitations. These drawbacks include:

1. *Lack of Trust*: Being programmatic in nature, techniques and models developed in such a way need to develop their trust before being fully accepted.
2. *Issue of Authenticity*: being synthetic, such data is often considered fake and not real. However, the increasing usage of synthetic data is becoming more accepted, but still models trained in such a way need to be validated on real world examples.
3. *Lack of Randomness*: real-world violent scenarios have a certain element of randomness in them. Such as particular human behaviours, backgrounds and existing objects which might be lacking in synthetic data. Moreover, real world fights can take many unexpected turns due to the environment in which the fight is happening, this element can sometimes be missing in synthetic data.
4. *Developer Bias:* this issue can still slither into synthetic data due to certain biases of the human developer who is designing the violent setups for execution.

## Ethics Statement

This research strictly adheres to ethical standards, prioritizing transparency, and integrity throughout the synthetic dataset generation process. The utilization of the modding tool in the Grand Theft Auto V (GTA-V) virtual environment is in accordance with principles of fair use within the modding community and aligns with guidelines set by relevant platforms.

The production of the data collected did not involve any human subjects, animal experimentation, nor any data from social media platforms. The authors have read and follow the ethical requirements for publication in Data in Brief.

## CRediT authorship contribution statement

**Muhammad Shahroz Nadeem:** Conceptualization, Data curation, Writing – original draft. **Fatih Kurugollu:** Supervision, Methodology, Writing – review & editing. **Hany F. Atlam:** Supervision, Writing – review & editing. **Virginia N.L. Franqueira:** Supervision, Validation, Resources, Project administration.

## Declaration of Competing Interest

The authors declare that they have no known competing financial interests or personal relationships that could have appeared to influence the work reported in this paper.

## Data Availability

Weapon Violence Dataset (Original data) (Kaggle).

## Appendix Supplementary materials

Image, application 1
